# Optimal Parameters of Deep Brain Stimulation in Essential Tremor: A Meta-Analysis and Novel Programming Strategy

**DOI:** 10.3390/jcm9061855

**Published:** 2020-06-14

**Authors:** I. Daria Bogdan, Teus van Laar, D.L. Marinus Oterdoom, Gea Drost, J. Marc C. van Dijk, Martijn Beudel

**Affiliations:** 1 Department of Neurology, University Medical Center Groningen, University of Groningen, 9713 GZ Groningen, The Netherlands; i.d.bogdan@umcg.nl (I.D.B.); g.drost@umcg.nl (G.D.); 2 Department of Neurosurgery, University Medical Center Groningen, University of Groningen, 9713 GZ Groningen, The Netherlands; d.l.m.oterdoom@umcg.nl (D.L.M.O.); j.m.c.van.dijk@umcg.nl (J.M.C.v.D.); 3 Department of Neurology, Amsterdam Neuroscience Institute, Amsterdam University Medical Center, 1007 MB Amsterdam, The Netherlands; m.beudel@amsterdamumc.nl

**Keywords:** DBS, tremor, stimulation parameters, DBS programming algorithm, DBS side effects, thalamic nucleus, zona incerta

## Abstract

The programming of deep brain stimulation (DBS) parameters for tremor is laborious and empirical. Despite extensive efforts, the end-result is often suboptimal. One reason for this is the poorly understood relationship between the stimulation parameters’ voltage, pulse width, and frequency. In this study, we aim to improve DBS programming for essential tremor (ET) by exploring a new strategy. At first, the role of the individual DBS parameters in tremor control was characterized using a meta-analysis documenting all the available parameters and tremor outcomes. In our novel programming strategy, we applied 10 random combinations of stimulation parameters in eight ET-DBS patients with suboptimal tremor control. Tremor severity was assessed using accelerometers and immediate and sustained patient-reported outcomes (PRO’s), including the occurrence of side-effects. The meta-analysis showed no substantial relationship between individual DBS parameters and tremor suppression. Nevertheless, with our novel programming strategy, a significantly improved (accelerometer *p* = 0.02, PRO *p* = 0.02) and sustained (*p* = 0.01) tremor suppression compared to baseline was achieved. Less side-effects were encountered compared to baseline. Our pilot data show that with this novel approach, tremor control can be improved in ET patients with suboptimal tremor control on DBS. In addition, this approach proved to have a beneficial effect on stimulation-related complications.

## 1. Introduction

Essential tremor (ET) is the most prevalent movement disorder, affecting up to 4% of the adult population [1]. Medical management of ET is limited and is often unsatisfactory [2]. For medically refractory cases, deep brain stimulation (DBS) may be considered. The long-term safety and efficacy of DBS are well-established [3], although it is not certain whether its initially reported superior tremor suppression is also achieved in successive cohorts [4]. Additionally, evidence shows that 33 out of 45 patients in one study (73.3%) reported waning tremor control at a mean time of 18.8 ± 15.1 months postoperatively [5].

The outcome of ET-DBS depends on several factors. Preoperative considerations include but are not limited to tremor characteristics [6] and the anatomical target of the intervention [7,8]. Postoperatively, optimal tremor reduction is achieved with time-consuming programming. The current strategy for symptom control starts with a standardized evaluation of several conventional stimulation parameters, representing the highest probability for success. Pulse widths with an estimated chronaxie (i.e., the minimum time for exciting a neural element using half the intensity to elicit a threshold response, for review see [9]) for myelinated axons in ET-DBS average 40–90 μs [10]. As far as frequency is concerned, it should be noted that during the first application of DBS, 50 Hz stimulation was considered as high-frequency stimulation [11]. Ever since, a broad range of stimulation frequencies up to 185 Hz has been explored, although there is no clear relation between the stimulation frequency and degree of tremor suppression. All parameters are further titrated in a ‘trial and error’ fashion, until satisfactory tremor suppression is achieved in the absence of side effects. In practice, this requires extensive programming sessions, in which patient fatigue may hamper achieving the desired results. Empirical titration becomes additionally challenging when conventional DBS parameters do not address individual requirements [12] or become subject to tremor habituation [13], requiring broader parameter searches than feasible. A clear understanding of the therapeutic role of the stimulation parameters is therefore essential. Unfortunately, the relation of any of the stimulation parameters and degree of tremor suppression remains insufficiently understood. A study exploring high-frequency stimulation as a putative cause for worsening balance in ET patients demonstrated that reducing stimulation frequency from 170–185 to 130 Hz after optimizing tremor control improved axial cerebellar signs [14]. In addition, therapeutic DBS intensity levels suppress tremor, while supra-therapeutic amplitudes and pulse-widths cause (gait) ataxia [15,16]. The deleterious effect of excessive stimulation translates thus into a narrow therapeutic window for tremor suppression.

Despite efforts to individualize and improve DBS programming [17,18], these are either not robust enough or are too technically challenging to be routinely applied in clinical practice. In the absence of an explicit, validated programming protocol, the process remains laborious and outcomes inconsistent, with average tremor reduction varying between 33 and 74% [2]. Outcomes may be improved with expert programming, shown to provide significant improvement in 37% of patients and partial improvement in 15% [12].

In this study, we aim to improve DBS programming protocols. We start by reviewing all documented DBS parameters and tremor outcomes in ET, with the aim to gain insight into the advancement of DBS programming over time, as well as to characterize the role of the DBS-parameters (voltage, pulse width, and frequency). Next, as a proof of concept, we introduce a novel approach for a timely and thorough exploration of the DBS parameter space in individual patients.

## 2. Methods

### 2.1. Meta-Analysis

#### 2.1.1. Search Strategy and Selection Criteria

We conducted a PubMed search for DBS parameters in ET on 05/03/2018, using the following string: (“DBS” OR “Deep Brain Stimulation”) AND (“essential tremor”). The search was conducted by two independent reviewers (DB and MO). Articles written in English, containing the key terms “DBS” or “stimulation” and “essential tremor” were included for full text screening. Animal studies, experiments in which patients had co-existing movement disorders (e.g., Parkinson’s disease), as well as review articles were excluded from further analysis. For full text screening the following exclusion criteria were applied: (1) either stimulation parameters or outcomes were not reported; (2) interventions following failed DBS or thalamotomy (the latter might still exert therapeutic effects); (3) reports earlier than three months post-operatively (when the microlesion effect might influence tremor) or with unspecified follow-up moment; (4) cohorts already reported; (5) irretrievable articles; (6) pooled data of ET with Parkinson’s patients; (7) IPG malfunctions. Review articles were checked for any relevant missing articles. The remaining articles were selected for data extraction.

#### 2.1.2. Data Extraction

From each relevant study, the following data were extracted: publication year, size of patient sample, DBS target, average stimulation voltage, pulse width and frequency, tremor reduction, scale of tremor measurement, follow-up moment and side-effects. Side-effects were categorized as paresthesia, gait and balance problems, ataxia, dysarthria and diplopia or visual disturbances.

#### 2.1.3. Data Synthesis

Data were recorded as mean ± standard deviation. For analysis, weighted values of the stimulation parameters and tremor outcomes were used. Reports of tremor suppression were heterogenous not only in terms of tremor scale, but also follow-up duration. Therefore, tremor outcomes were converted to percentages of tremor reduction and the primary outcome time point was used for each study. Given that the tremor measurements were performed at various follow-up durations, time might have possibly exerted an effect on the tremor outcomes owing to disease progression or tremor adaption. For this reason, follow-up durations were split into quartiles and the corresponding tremor outcomes were compared between the four groups.

#### 2.1.4. Comparisons

To characterize the explored range of stimulation parameters and the evolution of tremor outcomes from the first application of DBS for tremor (1987) until the present, we correlated these values with time, i.e., corresponding publication year. Next, we explored the relationship between voltage, pulse width, frequency and tremor control. We also documented the encountered side-effects and correlated their frequency with the corresponding stimulation parameters.

### 2.2. Experimental DBS Programming

#### 2.2.1. Patients

Eight consecutive patients treated with bilateral DBS for medically refractory ET participated in the study. Prior medical treatment included propranolol, primidone and topiramate, or a combination of the three. The efficacy of the treatment has been thoroughly reviewed before deeming the patient therapy-resistant and discussed multi-disciplinarily to establish the indication for DBS. All patients were implanted (Medtronic model 3387 lead, contacts 1.5 mm in length, spaced 3 mm center-to-center; Medtronic, Inc.) using the Leksell G frame (Elekta, Stockholm, Sweden). Accurate electrode positioning was tested using intra-operative macrostimulation (up to 5 V, with 60 µs pulses and a frequency of 185 Hz) and was confirmed by postoperative MRI (Philips Intera, Eindhoven, the Netherlands) and/or CT-scanning (Sensation 64, Siemens, Erlangen, Germany). The post-implantation CT images were fused with preoperative 3T-MRI images using BrainLAB software (BrainLAB, Heimstetten, Germany). The electrode configurations and stimulation parameters had been reviewed at length and optimized by using Medtronic’s 8840 N’Vision Clinician Programmer (Medtronic, Inc., Northridge, CA, USA), according to the best current practice [19]. None of the patients were on any medication for tremor following DBS. Patients were recruited at the outpatient neurological department at the University Medical Centre Groningen (UMCG). The experimental procedure was approved by the medical ethical review board of the UMCG (registration number 2017406) and deemed “care as usual”. Under these circumstances, written informed consent was not required.

#### 2.2.2. Experimental Paradigm

The experimental paradigm started with a baseline tremor measurement, using the current stimulation parameters. Tremor was assessed in various arm postures and quantified by an in-house developed accelerometer (UMCG, Groningen, The Netherlands). For each posture, the total distance amplitude (TDamp) was calculated over 5 s by a custom-written script in LabVIEW (2014 SP1) and was used to assess tremor severity. This was done by calculating the second integral of the accelerometer signal. After the baseline measurement, the posture in which tremor was most severe, was used for the adjustment of DBS parameters.

In this posture, 10 random combinations of stimulation parameters (voltage, pulse width and frequency) were tested. These combinations were generated by a custom-written script in MATLAB (version R2014a, MathWorks, MA, USA), for each patient individually. The ranges from which the stimulation parameters were randomly extracted were as follows: voltage 1.5–4 V with intervals of 0.1 V; pulse width 60–240 µs with intervals of 10µs and frequency 60–185 Hz with intervals of 5 Hz. The script selected *at random* one voltage, one pulse width and one frequency, from a pool of 12,844 theoretical combinations. The process was repeated 10 times for every patient. For each experimental combination, a 5s accelerometer recording was performed once the effects of the previous settings disappeared. Patients also indicated whether stimulation felt better in terms of tremor control compared to baseline. If side-effects emerged, stimulation was not further increased (thus attaining a pseudorandom set of parameters). However, the transition between combinations was done systematically to maximize the chance of employing a given set of parameters. Namely, we identified the parameters that needed to be lowered or increased in the subsequent setting. Given that higher current charges are more likely to cause side-effects, the parameters were prioritized as follows: parameter requiring the greatest decrements, followed by eventual lesser decrements, least increments and greatest increments, respectively. Therefore, parameters requiring decreases were adjusted first. If the remaining parameters needed to be increased, the one requiring the smallest increase was adjusted first. If the remaining changes were to cause side-effects, increments would be stopped, and the final combination would be noted. By applying this system, the pseudo character of the random parameters was deemed by the patient safety and not by the clinician’s bias.

In case the new stimulation parameters led to improved tremor control and/or less side-effects compared to the baseline settings, patients maintained these parameters. To evaluate whether the new empirical settings retained tremor control, patients were approached by telephone 6–17 weeks later. Patients indicated whether tremor control was similar, better or worse compared to baseline settings and whether side-effects had emerged.

#### 2.2.3. Evaluation of the Experimental Settings

The combinations of stimulation parameters that led to the best tremor reduction were identified by the individual ratings of the patients (subjective) and the accelerometer signal (objective). The effect on tremor of the best subjective and objective random stimulation parameters was compared to that of the baseline settings. Medium-term efficacy (i.e., beyond the clinical setting) was determined by contrasting the patient-reported improvements to baseline tremor control. Next, the subjective and objective stimulation parameters were compared to baseline settings to determine whether tremor control was achieved with significantly different parameters or levels of total electrical energy delivered (TEED, [20]).

## 3. Statistical Analysis

Statistical analysis was performed using SPSS (SPSS IBM version 23.0, Armonk, NY, USA). Data normality was tested with the Kolmogorov–Smirnov test. For correlations, Pearson’s correlation or Spearman’s rank-order correlation were used accordingly and reported together with the percentage of explained variance (r^2^). In the case of within-subject measurements, paired sample *t*-tests were used for normally distributed data and two-tailed Wilcoxon signed-rank tests for nonparametric distributions. For nonparametric, independent comparisons, the Kruskal–Wallis test was used. Significance was set at *p* < 0.05.

## 4. Results

### 4.1. Meta-Analysis

#### 4.1.1. Study Inclusion and Data Characteristics

**Figure 1:**
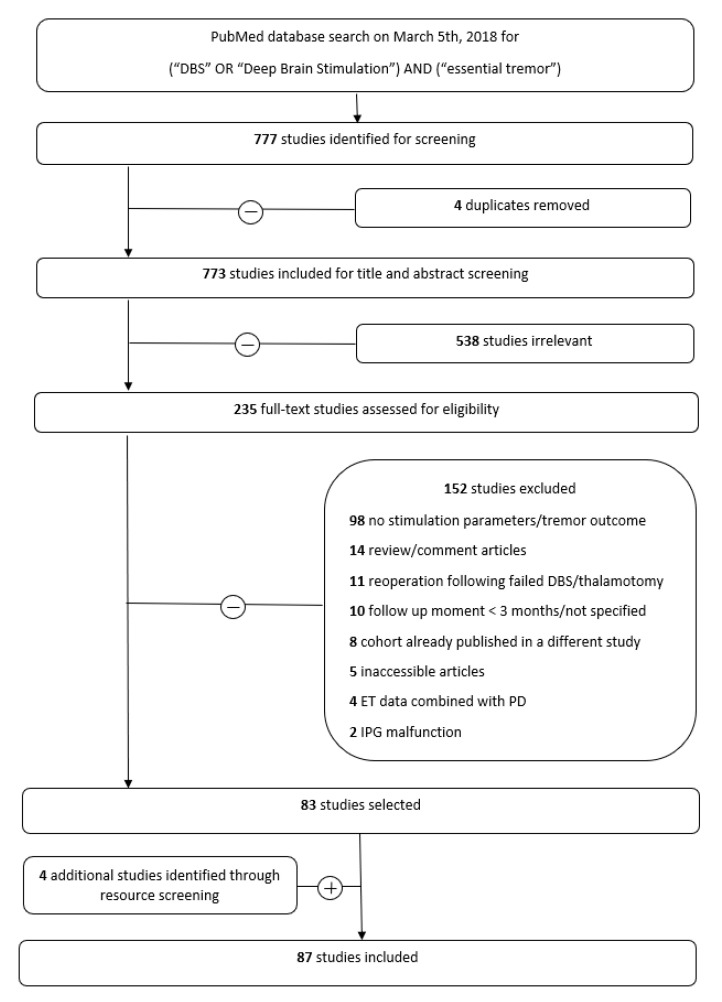
Preferred Reporting Items for Systematic Reviews and Meta-Analyses (PRISMA) flowchart.

Our PubMed search yielded a total of 777 studies. Four duplicates were removed, leaving 773 studies for title and abstract screening. Here, 538 studies did not fulfil the inclusion criteria. The remainder of 235 full-text studies were assessed for eligibility. Four additional studies were identified through cross-reference screening and added to the eighty-three studies selected for data extraction, involving 1652 patients (Figure 1, Supplementary Table S1). Data exhibited a nonparametric distribution.

#### 4.1.2. DBS parameters and Tremor Outcomes

The correlation coefficients of the stimulation parameters with time were r(1159) = −0.17, r^2^ = 0.02 (2.89%), *p* < 0.001 for voltage, r(1312) = −0.17, r^2^ = 0.02 (2.89%) *p* < 0.001 for pulse width and r(1258) = 0.08, r^2^ = 0.006 (0.6%) *p* < 0.05 for frequency. Overall, the average tremor suppression achieved by DBS was 62.98% ± 16%, r(1466) = 0.16, r^2^ = 0.02 (2.56%), *p* < 0.001 (Supplementary Figure S1).

#### 4.1.3. Correlation of Stimulation Parameters and Tremor Suppression

Follow-up times were split into quartiles, which yielded four groups of tremor outcomes corresponding to reports ranging from 3 to 6 months, 7 to 12 months, 13 to 30 months and 31 to 150 months, respectively. Although tremor outcomes differed significantly between the four groups (χ^2^(3) = 186, *p* < 0.001), the means did not exhibit a consistent downward trend (M_1_ = 57.42, 95% CI: 55.78–59.08; M_2_ = 70.93, 95% CI: 69.8–72.02; M_3_ = 62.25, 95% CI: 60.47–64.14; M_4_ = 55.95, 95% CI: 53.73–58.16). Thus, no correction for the variable follow-up times was applied. Regarding the interdependence of stimulation parameters and tremor, an inverse relationship is observed between voltage r(1159) = −0.09, r^2^ = 0.008 (.81%), *p* < 0.05, and tremor. Although the same trend is observed for pulse width r(1312) = −0.06, r^2^ = 0.003 (0.36%), *p* = 0.05, and frequency r(1258) = −0.03, r^2^ = 0.0009 (0.09%), *p* = 0.28, these are not statistically significant.

#### 4.1.4. Side Effects

Thirty-seven of the 87 included studies reported side effects (N = 785). Namely, 7.89% of patients complained of paresthesia (N = 62), 5.47% of gait and balance problems (N = 43), 2.03% of ataxia (N = 16), 15.28% of dysarthria (N = 120) and 0.12% of diplopia (N = 1). Only voltage exhibited a significant correlation, with dysarthria r(118) = 0.43, r^2^ = 0.43 (18.49%), *p* = 0.01.

### 4.2. Experimental DBS Programming

#### 4.2.1. Experimental Tremor Titration

**Figure 2:**
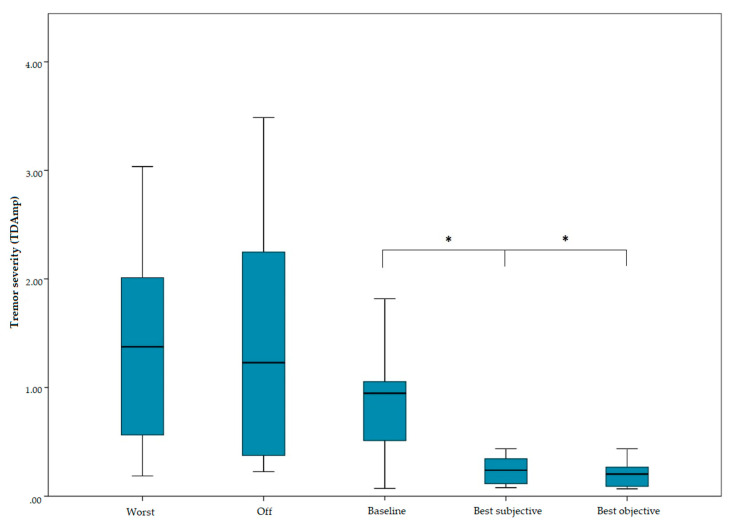
Experimental ET-DBS titration. Ten random combinations of stimulation parameters were tested and characterized subjectively (patient reported outcomes) and objectively (accelerometer measurements). The tremor outcomes during the experimental parameter exploration are categorized as follows: worst (least tremor reduction), off (stimulation turned off), baseline (initial settings), best subjective (best random settings according to the patient), best objective (best random settings according to accelerometer measurements). The novel programming strategy afforded significant tremor reduction (*t(6)* = −2.95, *p* = 0.02) in the absence of side-effects. (*) represents a statistically significant (*p* < 0.05) difference.

The experiment was conducted in eight ET patients (Table 1, Supplementary Figure S2). None of the patients was on any tremor medication following DBS surgery. Baseline tremor control could not be documented in one patient (ET6) and was excluded from the corresponding statistical analysis. Given that subjective improvement was nevertheless achieved and sustained in the thr medium term, ET6 is still documented in Table 1. The experimental paradigm showed significant tremor reduction compared to baseline stimulation, according to both subjective (*t(6)* = −2.95, *p* = 0.02) and objective (*t(6)* = −3.07, *p* = 0.02) measurements (Figure 2). Notably, the tremor improvement perceived by the patients corresponded to the accelerometer measurements. On average, the results were achieved after 23.8 ± 8.03 min per patient. Upon follow-up, 45% of the patients reported medium-term tremor control superior to baseline (*p* = 0.01). None of the patients reported the new setting as being worse than the previous. As far as side effects are concerned, four patients (ET2, ET4, ET7, ET8) reported stimulation-induced ataxia and/or dysarthria at baseline. Following experimental re-titration, side-effects were resolved in all but one patient (ET4), who reported re-emergence of ataxia upon follow-up.

#### 4.2.2. Random DBS Parameters

An example of the randomly generated parameters is given in Supplementary Figure S2. Parameter optimization was achieved with significantly broader pulse widths according to both subjective *Z* = −2.37, *p* = 0.01 and objective *Z* = −2.52, *p* = 0.01 measurements. Conversely, significantly lower frequencies proved superior to baseline settings in both subjective *Z* = −2.53, *p* = 0.01 and objective ratings *Z* = −2.52, *p* = 0.01. No significant difference was detected for either subjective *Z* = −1.12, *p* = 0.26 or objective *Z* = −1.54, *p* = 0.12 ratings of voltage. More optimal tremor reduction was achieved with higher levels of TEED, in both subjective *t(7)* = −2.12, *p* = 0.07 and objective *t(7)* = −3.08, *p* = 0.01 measurements.

## 5. Discussion

### 5.1. Meta-Analysis

Our meta-analysis shows that, in more than 30 years, the outcome of ET-DBS improved only modestly. Given that there was no relevant change in the studied DBS parameters, the observed improvement might possibly be attributed to improved patient selection [6], DBS targeting [7,21] and stereotactic planning [22]. Although beneficial, these developments are efficient mostly in the short-term. Provided that tremor outcomes significantly decline over time [23], with habituation of the stimulation settings accounting for more than 10% of the decrease in outcome [24], more efforts should be focused on programming. In line with this, there is evidence for alternating stimulation settings to reduce habituation. However, side-effects remain a great limiting factor.

From the available literature, there appears to be no substantial relationship between DBS parameters and tremor suppression or side-effects. Given that several follow-up reports extend up to 150 months, it raises the concern that tremor outcomes might be affected by either disease progression or tremor adaption. Although tremor outcomes differed significantly in the four time-groups (follow-up-duration quartiles), no downward trend suggesting declining outcomes was observed. As such, the observed differences stem more likely from patient heterogeneity, rather than being the effect of time. In line with these results, we introduced random combinations of stimulation parameters for conducting comprehensive and time-saving parameter searches in individual patients. We show that this novel programming strategy is effective in optimizing individual tremor control and resolving side effects.

Despite that DBS parameters remain relatively constant over time, the corresponding tremor outcomes show large variations. Although voltage is the only parameter to show a significant correlation with tremor suppression and dysarthria, the low value of the explained variation falls short to explain this observation (significance in this case reflects the large *N* rather than a clinical significance) These findings confirm the inconsistent results from DBS-parameter explorations reported elsewhere [25,26,27]. Such discrepancies suggest that uniform parameters might not exist, and that programming should particularly address the individual anatomy [7] and tremor characteristics [25].

#### The Rationale behind Conventional Stimulation Parameters

Conventional stimulation parameters have originally been extrapolated from structure–effect relationships to address the anatomical target and tremor characteristics [10,28]. However, they consistently appear to fall short of the mark, with room for improving clinical outcomes. Despite increasing evidence that better individualizing the DBS dose is key to maximizing symptom control [17,27], conventional parameters have remained the mainstay. However, as long as the precise spatiotemporal coordinates of the neuroanatomical substrate required for clinical benefits remain insufficiently understood, finding the optimal DBS parameters will remain challenging.

Firstly, the complexity of stimulating neural tissue stems from multiple determinants, e.g., the interaction with different neuronal elements and relative distance to the electrode [29,30], as well as the direction of propagation of the action potentials [31,32]. This is consistent with the difficulty of modeling electric field predictions [33,34]. Secondly, this spatial component of DBS is further raveled by the yet unknown mechanism of action. Several hypotheses have been proposed [35,36], illustrating the wide-ranging effects of DBS. Elucidating the precise effects that determine the clinical outcome will be key to refining the electric field predictions to address both the local and global dynamics of the targeted circuitopathies. Lastly, the third obstacle in finding optimal DBS parameters are the temporal adjustments of stimulation. Refining DBS to be delivered only in response to pathological biomarkers (adaptive DBS; aDBS) appears promising for both ameliorating symptoms and reducing side-effects [37,38].

Attempts to understand the stimulation parameters have yielded inconsistent results. Most commonly, the effect of varying one DBS-parameter is documented, while the remaining parameters are maintained at constant [39]. The limitation of such an approach is that the constant parameters determine the therapeutic window of the parameter of interest [16]. This generates irreproducible results due to inter-patient variability. In this study, we emphasize how insufficiently understood the interdependence of the stimulation parameters is, i.e., unexpected stimulation parameters can be clinically meaningful only when provided with the right interaction of these three parameters. Given that (1) the stimulation substrate exhibits a highly complex, dynamic and individualized spatiotemporal fabric and that (2) individual stimulation parameters cannot be considered for titration alone, future studies should allow for mutual dynamism to be exercised between the two. Therefore, programming strategies should be as robust as possible.

### 5.2. Experimental DBS Programming

Increasing understanding of the complexity of the stimulation substrate, as well as that of the interdependence of the stimulation parameters, has increasingly discouraged the use of conventional parameters. However, the infeasibility of clinically exploring the range of DBS parameters, to a greater extent, has precluded this transition. Testing ten random combinations of stimulation parameters results in a thorough, time-saving exploration, which allows for ET-DBS optimization in individual patients. Safety is ensured by gradually transitioning between combinations and having the patient report the emergence of side effects, beyond which stimulation is not further increased. Notably, the most optimal parameters are selected by patient intrinsic factors, e.g., anatomopathological substrate [40,41,42] and lead positioning [16,43]. The fact that significantly lower frequencies have been favored in this assumption-free trial is in line with the observed deleterious effects of supra-threshold frequency [14]. It is also tempting to attribute the resolution of side-effects in three of our four patients to this.This pilot approach raises thus great interest by opening the gate towards more individualized, comprehensive, and faster DBS titration.

The foremost limitations of the meta-analysis are publication bias [4] and perhaps overly enthusiastic early reports [44]. Additionally, the analysis of the relationship between DBS parameters and tremor outcomes could have benefited from objective tremor measurements (e.g., accelerometry). Nevertheless, validated tremor scales have been used [45]. Regarding the experiment, one limitation might be using shorter (between 1–2 min) wash-out periods than usual [18] in some patients. However, it has been shown that Vim-DBS for ET provides tremor suppression over seconds [2]. Additionally, the sustained tremor suppression upon follow-up excluded that the therapeutic benefit of the definitive experimental settings was confounded by phase-resetting or carryover effects. In addition, it would have been desirable to also provide tremor scores. However, the value of tremor scales would have been limited in this case, given that only one posture of one limb was assessed and tremor scales cannot detect subtle differences due to their ordinal character. Ideally, medium-term tremor reports should have been supplemented by accelerometer measurements. Another limitation might be using pre-determined electrode configurations. This was done because the electrode configurations with the largest therapeutic windows had already been determined in previous programming sessions and we wanted to limit patient fatigue to a minimum. However, it would be interesting, in a future study, to explore the role of random stimulation parameters in determining the therapeutic window. Lastly, there is no evidence that, after ten trials of random parameters, convergence is reached. However, we started with a pragmatic approach that could be tested during a regular outpatient visit. In future studies, we will explore whether testing more parameters brings further improvements.

## 6. Conclusions

DBS programming provides options beyond conventional parameter selection. Access to these parameters is particularly important for addressing ET that does not respond to conventional DBS parameters [12] or develops habituation [13,24]. Deep-learning modalities might be able to further refine this approach to avoid supra-therapeutic stimulation, minimize battery consumption, and enable the titration of more complex devices [46,47]. The role of the individual DBS parameters in tremor control remains elusive. Our proof of concept underscores the interdependence of voltage, pulse width, and frequency, warranting further research.

## Figures and Tables

**Table 1:** Patient demographics and deep brain stimulation (DBS) parameters. Baseline tremor control was measured prior re-titration with the patients retaining their current DBS settings. Next, ten random combinations of stimulation parameters were tested in eight essential tremor (ET) patients. The random combinations affording the most tremor suppression according to both patient-reported outcomes (subjective) and accelerometer recordings (objective) are presented here. Tremor severity is expressed in arbitrary units (TDAmp) as returned by the accelerometer. “Since youth” implies that the patients were diagnosed before the age of 18 years old.

Patient	Age (Years)	Gender	Disease Duration (years)	DBS Duration	DBS Target	DBS Contacts	Baseline				Best–Subjective				Best–Objective			
							V	P	F	TDAmp	V	P	F	TDAmp	V	P	F	TDAmp
ET1	53	male	Since youth	2	Vim	−1	2	90	185	0.87	3.2	180	80	0.32	3.3	200	95	0.20
ET3	77	male	22	1	Vim	−10	3	90	185	1.03	3.5	180	145	0.27	3.9	150	160	0.24
ET4	73	male	33	20	Vim	−0	0.8	60	180	0.14	1.6	70	165	0.11	2.1	170	85	0.11
ET5	69	male	52	1	Vim	−1/2+	2.8	60	185	0.07	2.7	210	170	0.07	3.2	140	175	0.07
Average tremor improvement (%) compared to baseline for Vim-DBS according to subjective (53%) and objective (58%) measurements.																		
ET2	78	male	Since youth	3	ZI	−3	3.3	60	185	0.94	3.6	60	160	0.36	3.9	70	150	0.28
ET6	70	male	27	4	ZI	−9/10+	1.7	60	180	n.a.	1.5	90	155	0.43	1.5	90	155	0.43
ET7	72	female	Since youth	6	ZI	−1	1.8	90	180	1.07	2.2	140	140	0.20	2,2	140	140	0.20
ET8	51	female	48	6	ZI	−1/2+	1	90	185	1.81	2.2	160	180	0.11	1.5	220	180	0.06
Average tremor improvement (%) compared to baseline for ZI-DBS according to subjective (79%) and objective (82%) measurements.																		

Abbreviations: ET = essential tremor, F = frequency (Hz), TDAmp = arbitrary unit of measurement representing tremor severity, P = pulse width (µs), V = voltage (V), Vim = ventral intermediate thalamic nucleus, ZI = zona incerta.

## Supplementary Materials

The following are available online at https://www.mdpi.com/2077-0383/9/6/1855/s1, Figure S1: Overall tremor outcomes; Figure S2. Experimental Set-up, Table S1. Characteristics of studies included in the meta-analysis.
