# Supplementary material for: Optimal Parameters of Deep Brain Stimulation in Essential Tremor: A Meta-Analysis and Novel Programming Strategy
Source: J Clin Med. 2020 Jun 14;9(6):1855. doi: 10.3390/jcm9061855 (PMC7356338; doi:10.3390/jcm9061855)
Supplement: Supplementary file 1 [file jcm-09-01855-s001.pdf]

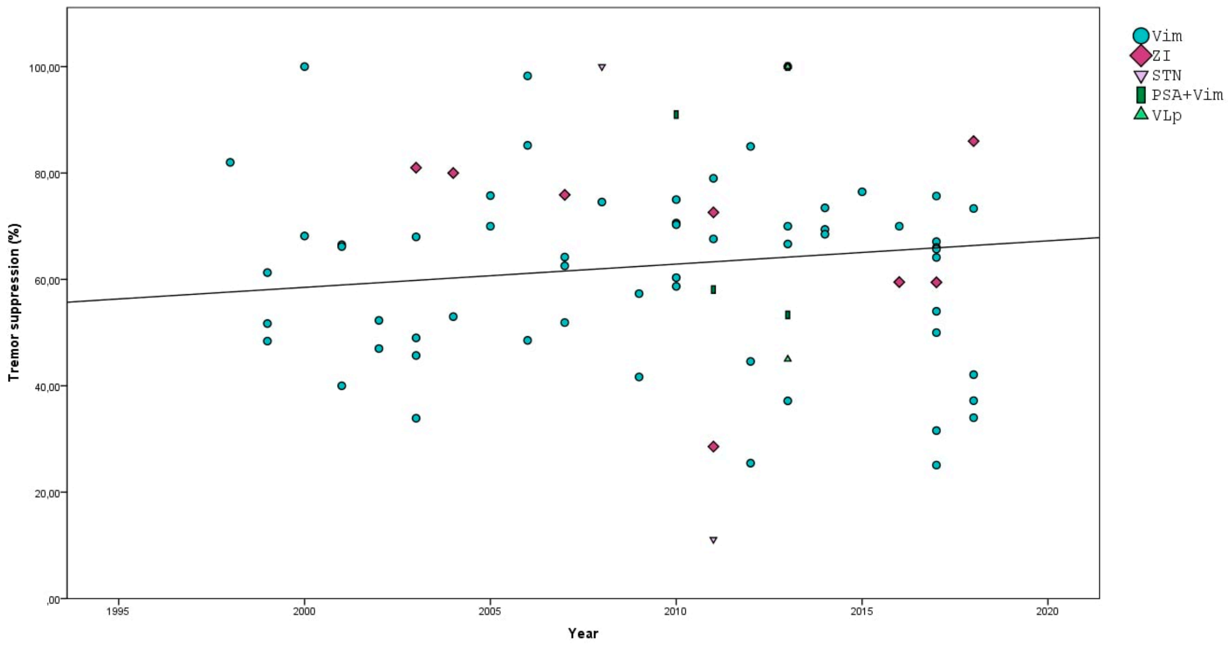

Supplementary Figure 1. Overall tremor outcomes. The average tremor suppression achieved with ET-DBS from 1987 until present was 62.98% ± 16%, r = 0.16, p < 0.001. Abbreviations: PSA = posterior subthalamic area, STN = subthalamic nucleus, Vim = ventral intermediate nucleus of the thalamus, VLp = ventrolateral thalamic nucleus, ZI = zona incerta.

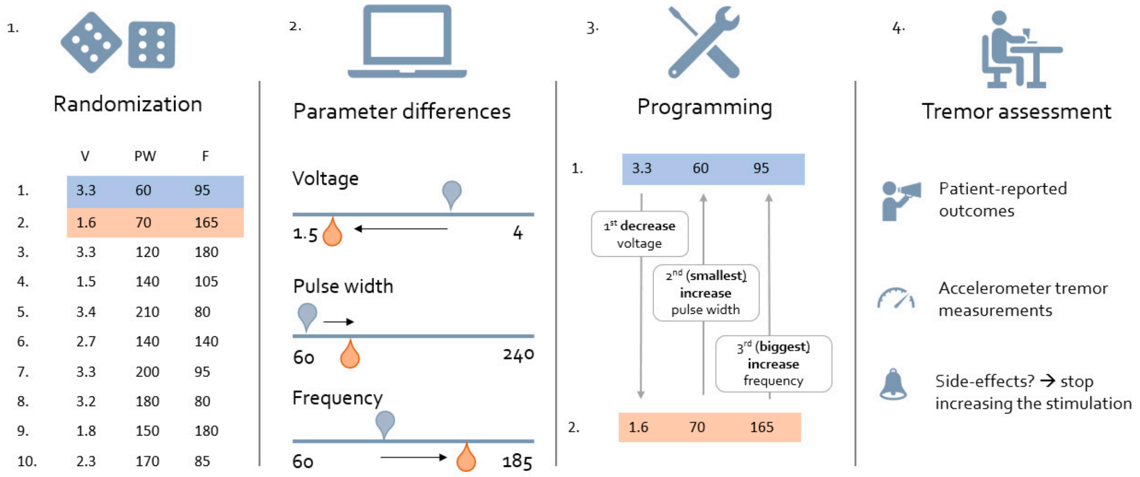

Supplementary Figure 2. Experimental Set-up. In this example, the successive steps for generating and testing the stimulation parameters for participant ET7 are depicted and described. 1. Randomization. The ranges for the 10 random values of voltage, pulse width and frequency were set, as following: voltage 1.5–4 V, pulse width 60–240 µs, and frequency 60–185 Hz. From here, 10 values for voltage, pulse width and frequency were generated at random, using a custom written MATLAB script. 2. Parameter differences. The transition between the 10 combinations was done systematically. Namely, (i) the parameters that needed to be lowered were identified and adjusted first; (ii) if the remaining parameters needed to be increased, the one requiring the smallest increase was adjusted next, followed by the one requiring the bigger increase. For example, let us consider the transition between the first combination, depicted in blue, and the second, in orange. The adjustments needed to transition from the blue to the orange combinations can be summarized as follows:

voltage needs to be lowered and both pulse width and frequency need to be increased. 3. Programming. Given that parameters needing to be lowered are the first to be adjusted, the voltage was changed first. Second, we considered the remaining parameters, which both needed to be increased (in this case, pulse width and frequency). Pulse width needed to be increased from 60 to 70 μs, which required one increment (1 × 10 μs). Frequency needed to be increased from 95 to 165 Hz, requiring 14 increments (14 × 5 Hz). Thus, the parameter requiring the smallest increase, in this case pulse width, was changed first followed by the bigger increase, in this case, the frequency. 4. Tremor assessment. For each combination, tremor was rated by patients, as well as objectively quantified with accelerometry. During the entire process, patients were requested to report the emergence of side effects, beyond which stimulation would not be further increased. This process was iterated 10 times in each patient. Abbreviations: V = voltage, PW = pulse width, F = frequency.

Supplementary Table 1. Characteristics of studies included in the meta-analysis.

References	*N*	DBS target	Voltage ± SD	Pulse width ± SD	Frequency ± SD	Follow up ± SD	Tremor reduction (%)
Benabid et al., 1987 [1]	6	Vim	n.a.	n.a.	130	n.a.	n.a.
Blond et al., 1992 [2]	4	Vim	n.a.	n.a.	130	11 ± 5	n.a.
Ondo et al., 1998 [3]	12	Vim	2.7 ± 0.9	256.4 ± 91.6	168.9 ± 16.4	3	82
Hariz et al., 1999 [4]	36	Vim	1.9 ± 0.74	73.3 ± 30	163.7 ± 17.6	3	48.4
Limousin et al., 1999 [5]	37	Vim	2.17 ± 0.81	92.73 ± 39.26	156.36 ± 24.72	3	51.7
Kumar et al., 1999 [6]	9	Vim	2 ± 1	195 ± 75	115 ± 15	14.9 ± 8.1	61.28
Obwegeser et al., 2000 [7]	27	Vim	n.a.	n.a.	n.a.	n.a.	68.17
Schuurman et al., 2000 [8]	7	Vim	n.a.	n.a.	n.a.	n.a.	100
Ceballos-Baumann et al., 2001 [9]	6	Vim	2.53 ± 0.76	115 ± 74.49	155.83 ± 25.18	min 4	66.53
Koller et al., 2001 [10]	25	Vim	3.4 ± 0.7	78.8 ± 37.3	157.5 ± 28.2	3	40
Ondo et al., 2001 [11]	11	Vim	2.6	212	149.5	3	66.17
Fields et al., 2002 [12]	40	Vim	3.32 ± 0.65	86.57 ± 24.33	150.07 ± 18.56	3	52.29
Hariz et al., 2002 [13]	27	Vim	n.a.	n.a.	n.a.	12.5 ± 4.7	47
Bryant et al., 2003 [14]	16	Vim	2.74 ± 0.99	119 ± 44	160 ± 28.2	13	33.9
Kumar et al., 2003 [15]	5	Vim	n.a.	n.a.	n.a.	12	68
Murata et al., 2003 [16]	8	Zi	1.99 ± 0.9	60	130	6	81
Rehncrona et al., 2003 [17]	19	Vim	2.4 ± 0.9	91.6 ± 33.9	171.6 ± 19.2	24	49
Sydow et al., 2003 [18]	19	Vim	2.3 ± 0.9	85.9 ± 28.2	163.2 ± 24.1	12	45.69
Vaillancourt et al., 2003 [19]	6	Vim	2.9 ± 1.5	80 ± 24.5	185	min 3	n.a.
Woods et al., 2003 [20]	49	Vim	3.34 ± 0.64	92.23 ± 32.26	148.05 ± 25.46	n.a.	n.a.
Papavassiliou et al., 2004 [21]	37	Vim	2.7 ± 0.9	98.5 ± 27	184 ± 7.5	26	53
Plaha et al., 2004 [22]	4	Zi	1.8 ± 0.2	108.8 ± 14.4	170 ± 11.5	12	80
Ushe et al., 2004 [23]	16	Vim	3.4 ± 0.5	67.5 ± 13.4	185	30.3 ± 17.8	n.a.
Capelle et al., 2005 [24]	2	Vim	2.8 ± 0.25	180 ± 51.96	146.66 ± 12.58	48	n.a.
Lee and Kondziolka, 2005 [25]	18	Vim	n.a.	n.a.	n.a.	27 ± 10	75.75
Putzke et al., 2005 [26]	22	Vim	2.8 ± 1.1	80.9 ± 33	158.3 ± 30.8	3	70
Chen et al., 2006 [27]	13	Vim	3.78 ± 1.13	110.76 ± 57.94	181.92 ± 11.09	20.48 ± 16.97	n.a.
Kronenbuerger et al., 2006 [28]	4	Vim	n.a.	n.a.	n.a.	3	48.54
Kuncel et al., 2006 [29]	9	Vim	2.9 ± 0.86	87.85 ± 21.9	150.35 ± 24.21	20.86 ± 17.62	98.26
Pahwa et al., 2006 [30]	22	Vim	3.26 ± 0.67	111	155	3	n.a.

	22	Vim	3.55 ± 1.09	111	155	60	50.42
Ushe et al., 2006 [31]	11	Vim	3.3 ± 0.5	70 ± 15	185	min 4	85.2
Blomstedt et al., 2007 [32]	19	Vim	1.8 ± 0.7	68 ± 14	164 ± 15	13 ± 4.9	51.89
Earheart et al., 2007 [33]	11	Vim	3.7 ± 0.62	81.8 ± 23.6	185	47.6 ± 26.4	62.55
Herzog et al., 2007 [34]	10	Vim	2.4 ± 0.76	67 ± 13.11	141.76 ± 20.07	14.14 ± 10	64.2
Plaha et al., 2007 [35]	6	Zi	2.48 ± 1.04	120 ± 42.42	147.14 ± 25.63	57.6	75.9
Lind et al., 2008 [36]	3	STN	1.76 ± 0.7	90	131.6 ± 2.88	104	100
Pilitsis et al., 2008 [37]	28	Vim	3.21 ± 1.78	61.6 ± 9.43	177.3 ± 18.55	40 ± 17	74.55
Earheart et al., 2009 [38]	13	Vim	3.6 ± 0.9	87.8 ± 31.1	181 ± 13.9	50.8 ± 30.6	41.66
Kronenbuerger et al., 2009 [39]	12	Vim	2.7 ± 0.9	98.2 ± 55.5	145.9 ± 16.7	n.a.	57.33
De Los Reyes et al., 2010 [40]	1	Vim	2.6	90	185	24	75
DiLorenzo et al., 2010 [41]	1	Vim	2.8	60	130	144	n.a.
Fasano et al., 2010 [42]	11	Vim	n.a.	n.a.	n.a.	24.7 ± 20.3	70.6
Fytagoridis et al., 2010 [43]	27	PSA	2.2 ± 0.9	65 ± 11	164 ± 21	12	91
Graff-Radford et al., 2010 [44]	31	Vim	2.8	91	151	6	60.33
Sitburana et al., 2010 [45]	11	Vim	3 ± 0.8	60	170	6	58.7
Zhang et al., 2010 [46]	35	Vim	2.89 ± 0.74	93.4 ± 20.3	173.7 ± 21.2	24 ± 12	n.a.
Barbe et al., 2011 [47]	23	Vim	2.4 ± 1.01	66.97 ± 21.55	133.72 ± 14.6	49 ± 39	67.61
Blomstedt[1] et al., 2011 [48]	1	Zi	0.9	60	185	3	28.57
	2	STN	2.45 ± 0.05	60	165 ± 28.28	3	11.11
Blomstedt[2] et al., 2011 [49]	15	Vim	n.a.	n.a.	n.a.	28 ± 24	55.33
	34	PSA	n.a.	n.a.	n.a.	12	58.09
Mandat et al., 2011 [50]	18	Vim	1.75 ± 1.06	80	130	3	79
Plaha et al., 2011 [51]	15	Zi	2.65 ± 1.2	105 ± 16	150 ± 22.9	48	72.6
de Oliveira et al., 2012 [52]	26	Vim	n.a.	n.a.	n.a.	57.14 ± 30	25.45
Favilla et al., 2012 [53]	28	Vim	n.a.	n.a.	n.a.	6	44.58
Nazzaro et al., 2012 [54]	78	Vim	n.a.	n.a.	n.a.	12	85
Chang et al., 2013 [55]	1	Vim	n.a.	n.a.	n.a.	6	100
	1	PSA	n.a.	n.a.	n.a.	6	100
	2	PSA + Vim	n.a.	n.a.	n.a.	6	100
Shih et al., 2013 [56]	12	Vim	2.7 ± 0.8	78 ± 28.9	155.5 ± 26.9	55.9 ± 34.4	n.a.
	33	Vim	3.1 ± 0.7	112 ± 47.8	184 ± 1.9	55.9 ± 34.4	n.a.
Baizabal-Carvallo et al., 2013 [57]	13	Vim	3.25 ± 0.81	114 ± 41.9	161.7 ± 23.9	132.54 ± 15.3	37.16
Cagnan et al., 2013 [58]	10	Vim	2.43 ± 0.67	78 ± 15.49	145 ± 24.15	6	70

Pedrosa et al., 2013 [59]	16	Vim	2.47 ± 1.11	76.88 ± 23.8	128.13 ± 8.54	50.4 ± 37.2	n.a.
Ramirez-Zamora et al., 2013 [60]	1	Vim	3.5	90	185	3	n.a.
Børretzen et al., 2014 [61]	30	Vim	3.5 ± 1.2	90 ± 30	181 ± 50	66 ± 30	n.a.
Coenen et al., 2014 [62]	11	Vim	3 ± 1.4	76 ± 16	173 ± 12	9 ± 6	69.4
Ehlen et al., 2014 [63]	13	Vim	3.1 ± 1.43	70.75 ± 14.98	149.74 ± 32.75	36.24 ± 33.96	68.5
Groppa et al., 2014 [64]	7	Vim	2.4 ± 0.6	62.5 ± 8.6	134.2 ± 14.4	19.7 ± 14.4	73.45
Mücke et al., 2014 [65]	16	PSA + Vim	2.39 ± 0.96	64.01 ± 10.61	130.25 ± 7.9	3	n.a.
Pedrosa et al., 2014 [66]	17	VLp	2.46 ± 0.96	75.5 ± 26	129 ± 8.61	55.2 ± 38.4	n.a.
Huss et al., 2015 [67]	70	Vim	n.a.	n.a.	n.a.	13 ± 10	76.47
Becker et al., 2016 [68]	16	PSA + Vim	2.6 ± 1.8	75 ± 15.5	130.6 ± 19.56	35.4 ± 15.5	n.a.
Ehlen et al., 2016 [69]	13	Vim	3.16 ± 1.47	60	157.5 ± 55.63	42 ± 38.28	n.a.
Fytagoridis et al., 2016 [70]	50	Zi	2.1 ± 0.8	64.6 ± 15.6	160.6 ± 21.6	12	59.5
Gibson et al., 2016 [71]	10	Vim	1.74 ± 0.6	60	135 ± 15.81	3	70.3
Ramirez-Zamora et al., 2016 [60]	4	Vim	2.35 ± 0.35	82.5 ± 15	181.25 ± 7.5	12 ± 5.71	n.a.
Reich et al., 2016 [72]	10	Vim	2.27 ± 0.76	61.65 ± 7.99	157.5 ± 21.68	24 ± 7	70
Allert et al., 2017 [73]	1	Vim	5.25 ± 0.25	90	130	12	n.a.
Cagnan et al., 2017 [74]	6	Vim	2.15 ± 0.65	84.28 ± 52.23	152.14 ± 27.66	6	n.a.
Chen et al., 2017 [75]	44	Vim	2.2 ± 0.7	72.45 ± 17.65	170.55 ± 21.3	12	67.1
Chockalingam et al., 2017 [76]	13	Vim	n.a.	n.a.	n.a.	min 3	25.09
Cury et al., 2017 [77]	35	Vim	n.a.	n.a.	n.a.	12	66
Ehlen et al., 2017 [78]	13	Vim	3.1 ± 1.39	60	146.92 ± 31.66	34.08 ± 28.08	64.13
Fiechter et al., 2017 [79]	7	Vim	3 ± 0.7	180 ± 40	141.5 ± 19.7	13.5 ± 8.5	54
Haddad et al., 2017 [80]	16	Vim	2.41 ± 0.48	67.6 ± 10.96	133 ± 12.64	57 ± 21	65.7
Holslag et al., 2017 [81]	19	Vim	2.4 ± 1	73 ± 15	184 ± 1.6	127.2 ± 54	31.57
	25	Zi	2.4 ± 0.9	70 ± 17	177 ± 19	69.6 ± 36	59.45
King et al., 2017 [82]	20	Vim	2.6 ± 0.9	66 ± 13	155 ± 27	12	min 50
Wharen et al., 2017 [83]	127	Vim	2.58 ± 1.73	96	164 ± 44	12	75.68
Akram et al., 2018 [84]	5	Vim	2.2	60	154 ± 25	23.6 ± 12	34
De Jesus et al., 2018 [85]	11	Vim	2.74 ± 0.76	100 ± 25	156.81 ± 24.31	41.54 ± 41.71	42.1
Mücke et al., 2018 [86]	12	Vim	2.3 ± 1.3	66.25 ± 12.44	128.75 ± 10.45	60.92 ± 12.31	73.33

Abbreviations: n.a. = not available; PSA = posterior subthalamic area; STN = subthalamic nucleus; Vim = ventral intermediate thalamic nucleus; VLp = ventrolateral thalamic nucleus.

Reference

1. Benabid, A.L.; Pollak, P.; Louveau, A.; Henry, S.; De rougemont, J. Combined (thalamotomy and stimulation) stereotactic surgery of the VIM thalamic nucleus for bilateral Parkinson disease. *Appl. Neurophysiol.* **1987**, *50*, 344–346.
2. Blond, S.; Caparros-lefebvre, D.; Parker, F.; Assaker, R.; Petit, H.; Guieu, J.D., Christiaens, J.L. Control of tremor and involuntary movement disorders by chronic stereotactic stimulation of the ventral intermediate thalamic nucleus. *J. Neurosurg.* **1992**, *77*, 62–68.
3. Ondo, W.; Jankovic, J.; Schwartz, K.; Almaguer, M.; Simpson, R.K. Unilateral thalamic deep brain stimulation for refractory essential tremor and Parkinson's disease tremor. *Neurology* **1998**, *51*, 1063–1069.
4. Hariz, M.I.; Shamsgovara, P.; Johansson, F.; Hariz, G.; Fodstad, H. Tolerance and tremor rebound following long-term chronic thalamic stimulation for Parkinsonian and essential tremor. *Stereotact Funct. Neurosurg.* **1999**, *72*, 208–218.
5. Limousin, P.; Speelman, J.D.; Gielen, F.; Janssens, M. Multicentre European study of thalamic stimulation in parkinsonian and essential tremor. *J. Neurol. Neurosurg. Psychiatry.* **1999**, *66*, 289–296.
6. Kumar, K.; Kelly, M.; Toth, C. Deep brain stimulation of the ventral intermediate nucleus of the thalamus for control of tremors in Parkinson's disease and essential tremor. *Stereotact Funct. Neurosurg.* **1999**, *72*, 47–61.
7. Obwegeser, A.A.; Uitti, R.J.; Turk, M.F.; Strongosky, A.J.; Wharen, R.E. Thalamic stimulation for the treatment of midline tremors in essential tremor patients. *Neurology* **2000**, *54*, 2342–2344.
8. Schuurman, P.R.; Bosch, D.A.; Bossuyt, P.M.; Bonsel, G.J.; van Someren, E.J.; de Bie, R.M.; Merkus, M.P.; Speelman, J.D. A comparison of continuous thalamic stimulation and thalamotomy for suppression of severe tremor. *N. Engl. J. Med.* **2000**, *342*, 461–468.
9. Ceballos-baumann, A.O.; Boecker, H.; Fogel, W.; Alesch, F.; Bartenstein, P.; Conrad, B.; Diederich, N.; von Falkenhayn, I.; Moringlane, J.R.; Schwaiger, M.; et al. Thalamic stimulation for essential tremor activates motor and deactivates vestibular cortex. *Neurology* **2001**, *56*, 1347–1354.
10. Koller, W.C.; Lyons, K.E.; Wilkinson, S.B.; Troster, A.I.; Pahwa, R. Long-term safety and efficacy of unilateral deep brain stimulation of the thalamus in essential tremor. *Mov. Disord.* **2001**, *16*, 464–468.
11. Ondo, W.; Almaguer, M.; Jankovic, J.; Simpson, R.K. Thalamic deep brain stimulation: Comparison between unilateral and bilateral placement. *Arch. Neurol.* **2001**, *58*, 218–222.
12. Fields, J.A.; Tröster, A.I.; Woods, S.P.; Higginson, C.I.; Wilkinson, S.B.; Lyons, K.E.; Koller, W.C.; Pahwa, R. Neuropsychological and quality of life outcomes 12 months after unilateral thalamic stimulation for essential tremor. *J. Neurol. Neurosurg. Psychiatry.* **2003**, *74*, 305–311.
13. Hariz, G.M.; Lindberg, M.; Bergenheim, A.T. Impact of thalamic deep brain stimulation on disability and health-related quality of life in patients with essential tremor. *J. Neurol. Neurosurg. Psychiatry.* **2002**, *72*, 47–52.
14. Bryant, J.A.; De salles, A.; Cabatan, C.; Frysinger, R.; Behnke, E.; Bronstein, J. The impact of thalamic stimulation on activities of daily living for essential tremor. *Surg. Neurol.* **2003**, *59*, 479–484.
15. Kumar, R.; Lozano, A.M.; Sime, E.; Lang, A.E. Long-term follow-up of thalamic deep brain stimulation for essential and parkinsonian tremor. *Neurology* **2003**, *61*, 1601–1604.
16. Murata, J.; Kitagawa, M.; Uesugi, H.; Saito, H.; Iwasaki, Y.; Kikuchi, S.; Tashiro, K.; Sawamura, Y. Electrical stimulation of the posterior subthalamic area for the treatment of intractable proximal tremor. *J. Neurosurg.* **2003**, *99*, 708–715.
17. Rehncrona, S.; Johnels, B.; Widner, H.; Törnqvist, A.L.; Hariz, M.; Sydow, O. Long-term efficacy of thalamic deep brain stimulation for tremor: Double-blind assessments. *Mov. Disord.* **2003**, *18*, 163–170.
18. Sydow, O.; Thobois, S.; Alesch, F.; Speelman, J.D. Multicentre European study of thalamic stimulation in essential tremor: A six year follow up. *J. Neurol. Neurosurg. Psychiatry* **2003**, *74*, 1387–1391.
19. Vaillancourt, D.E.; Sturman, M.M.; Verhagen metman, L.; Bakay, R.A.; Corcos, D.M. Deep brain stimulation of the VIM thalamic nucleus modifies several features of essential tremor. *Neurology* **2003**, *61*, 919–925.
20. Woods, S.P.; Fields, J.A.; Lyons, K.E.; Pahwa, R.; Tröster, A.I. Pulse width is associated with cognitive decline after thalamic stimulation for essential tremor. *Parkinsonism Relat Disord.* **2003**, *9*, 295–300.
21. Papavassiliou, E.; Rau, G.; Heath, S.; Abosch, A.; Barbaro, N.M.; Larson, P.S.; Lamborn, K.; Starr, P.A. Thalamic deep brain stimulation for essential tremor: Relation of lead location to outcome. *Neurosurgery* **2004**, *54*, 1120–1129.

22. Plaha, P.; Patel, N.K.; Gill, S.S. Stimulation of the subthalamic region for essential tremor. *J. Neurosurg.* **2004**, *101*, 48–54.
23. Ushe, M.; Mink, J.W.; Revilla, F.J.; Wernle, A.; Schneider Gibson, P.; McGee-Minnich, L.; Hong, M.; Rich, K.M.; Lyons, K.E.; Pahwa, R.; et al. Effect of stimulation frequency on tremor suppression in essential tremor. *Mov. Disord.* **2004**, *19*, 1163–1168.
24. Capelle, H.H.; Simpson, R.K.; Kronenbuerger, M.; Michaelsen, J.; Tronnier, V.; Krauss, J.K. Long-term deep brain stimulation in elderly patients with cardiac pacemakers. *J. Neurosurg.* **2005**, *102*, 53–59.
25. Lee, J.Y.; Kondziolka, D. Thalamic deep brain stimulation for management of essential tremor. *J. Neurosurg.* **2005**, *103*, 400–403.
26. Putzke, J.D.; Whaley, N.R.; Baba, Y.; Wszolek, Z.K.; Uitti, R.J. Essential tremor: Predictors of disease progression in a clinical cohort. *J. Neurol. Neurosurg. Psychiatry* **2006**, *77*, 1235–1237.
27. Chen, H.; Hua, S.E.; Smith, M.A.; Lenz, F.A.; Shadmehr, R. Effects of human cerebellar thalamus disruption on adaptive control of reaching. *CEREB Cortex*. **2006**, *16*, 1462–1473.
28. Kronenbuerger, M.; Fromm, C.; Block, F.; Coenen, V.A.; Rohde, I.; Rohde, V.; Noth, J. On-demand deep brain stimulation for essential tremor: A report on four cases. *Mov. Disord.* **2006**, *21*, 401–405.
29. Kuncel, A.M.; Cooper, S.E.; Wolgamuth, B.R.; Clyde, M.A.; Snyder, S.A.; Montgomery Jr., E.B.; Rezai, A.R.; Grill, W.M. Clinical response to varying the stimulus parameters in deep brain stimulation for essential tremor. *Mov. Disord.* **2006**, *21*, 1920–1928.
30. Pahwa, R.; Lyons, K.E.; Wilkinson, S.B.; Simpson Jr., R.K.; Ondo, W.G.; Tarsy, D.; Norregaard, T.; Hubble, J.P.; Smith, D.A.; Hauser, R.A.; et al. Long-term evaluation of deep brain stimulation of the thalamus. *J. Neurosurg.* **2006**, *104*, 506–512.
31. Ushe, M.; Mink, J.W.; Tabbal, S.D.; Hong, M.; Schneider Gibson, P.; Rich, K.M.; Lyons, K.E.; Pahwa, R.; Perlmutter, J.S. Postural tremor suppression is dependent on thalamic stimulation frequency. *Mov. Disord.* **2006**, *21*, 1290–1292.
32. Blomstedt, P.; Hariz, G.M.; Hariz, M.I.; Koskinen, L.O. Thalamic deep brain stimulation in the treatment of essential tremor: A long-term follow-up. *Br. J. Neurosurg.* **2007**, *21*, 504–509.
33. Earhart, G.M.; Hong, M.; Tabbal, S.D.; Perlmutter, J.S. Effects of thalamic stimulation frequency on intention and postural tremor. *Exp. Neurol.* **2007**, *208*, 257–263.
34. Herzog, J.; Hamel, W.; Wenzelburger, R.; Pötter, M.; Pinsker, M.O.; Bartussek, J.; Morsnowski, A.; Steigerwald, F.; Deuschl, G.; Volkmann, J. Kinematic analysis of thalamic versus subthalamic neurostimulation in postural and intention tremor. *Brain* **2007**, *130*, 1608–1625.
35. Plaha, P.; Khan, S.; Gill, S.S. Bilateral stimulation of the caudal zona incerta nucleus for tremor control. *J. Neurol. Neurosurg. Psychiatry* **2008**, *79*, 504–513.
36. Lind, G.; Schechtmann, G.; Lind, C.; Winter, J.; Meyerson, B.A.; Linderoth, B. Subthalamic stimulation for essential tremor. Short- and long-term results and critical target area. *Stereotact Funct. Neurosurg.* **2008**, *86*, 253–258.
37. Pilitsis, J.G.; Metman, L.V.; Toleikis, J.R.; Hughes, L.E.; Sani, S.B.; Bakay, R.A. Factors involved in long-term efficacy of deep brain stimulation of the thalamus for essential tremor. *J. Neurosurg.* **2008**, *109*, 640–646.
38. Earhart, G.M.; Clark, B.R.; Tabbal, S.D.; Perlmutter, J.S. Gait and balance in essential tremor: Variable effects of bilateral thalamic stimulation. *Mov. Disord.* **2009**, *24*, 386–391.
39. Kronenbuerger, M.; Konczak, J.; Ziegler, W.; Buderath, P.; Frank, B.; Coenen, V.A.; Kiening, K.; Reinacher, P.; Noth, J.; Timmann, D. Balance and motor speech impairment in essential tremor. *Cerebellum* **2009**, *8*, 389–398.
40. De los reyes, K.; Chandrasekhar, S.S.; Tagliati, M.; Alterman, R. Successful implantation of a deep brain stimulator for essential tremor in a patient with a preexisting cochlear implant: Surgical technique: Technical case report. *Neurosurgery* **2010**, *66*, 372.
41. Dilorenzo, D.J.; Jankovic, J.; Simpson, R.K.; Takei, H.; Powell, S.Z. Long-term deep brain stimulation for essential tremor: 12-year clinicopathologic follow-up. *Mov. Disord.* **2010**, *25*, 232–238.
42. Fasano, A.; Herzog, J.; Raethjen, J.; Rose, F.E.; Muthuraman, M.; Volkmann, J.; Falk, D.; Elble, R.; Deuschl, G. Gait ataxia in essential tremor is differentially modulated by thalamic stimulation. *Brain* **2010**, *133*, 3635–3648.
43. Fytagoridis, A.; Blomstedt, P. Complications and side effects of deep brain stimulation in the posterior subthalamic area. *Stereotact Funct. Neurosurg.* **2010**, *88*, 88–93.

44. Graff-radford, J.; Foote, K.D.; Mikos, A.E.; Bowers, D.; Fernandez, H.H.; Rosado, C.A.; Rodriguez, R.L.; Malaty, I.A.; Haq, I.U.; Jacobson, C.E.; et al. Mood and motor effects of thalamic deep brain stimulation surgery for essential tremor. *Eur. J. Neurol.* **2010**, *17*, 1040–1046.
45. Sitburana, O.; Almaguer, M.; Ondo, W.G. A pilot study: Microlesion effects and tremor outcome in the ventrointermediate deep brain stimulation (VIM-DBS). *Clin. Neurol. Neurosurg.* **2010**, *112*, 106–109.
46. Zhang, K.; Bhatia, S.; Oh, M.Y.; Cohen, D.; Angle, C.; Whiting, D. Long-term results of thalamic deep brain stimulation for essential tremor. *J. Neurosurg.* **2010**, *112*, 1271–1276.
47. Barbe, M.T.; Liebhart, L.; Runge, M.; Pauls KA, M.; Wojtecki, L.; Schnitzler, A.; Allert, N.; Fink, G.; Sturm, V.; Maarouf, M.; et al. Deep brain stimulation in the nucleus ventralis intermedius in patients with essential tremor: Habituation of tremor suppression. *J. Neurol.* **2011**, *258*, 434–439.
48. Blomstedt, P.; Sandvik, U.; Linder, J.; Fredricks, A.; Forsgren, L.; Hariz, M.I. Deep brain stimulation of the subthalamic nucleus versus the zona incerta in the treatment of essential tremor. *Acta Neurochir.* **2011**, *153*, 2329–2335.
49. Blomstedt, P.; Sandvik, U.; Hariz, M.I.; Fytagoridis, A.; Forsgren, L.; Hariz, G.M.; Koskinen, L.O.D. Influence of age, gender and severity of tremor on outcome after thalamic and subthalamic DBS for essential tremor. *Parkinsonism Relat. Disord.* **2011**, *17*, 617–620.
50. Mandat, T.; Koziara, H.; Rola, R.; Bonicki, W.; Nauman, P. Thalamic deep brain stimulation in the treatment of essential tremor. *Neurol. Neurochir. Pol.* **2011**, *45*, 37–41.
51. Plaha, P.; Javed, S.; Agombar, D.; O' Farrell, G.; Khan, S.; Whone, A.; Gill, S. Bilateral caudal zona incerta nucleus stimulation for essential tremor: Outcome and quality of life. *J. Neurol. Neurosurg. Psychiatry* **2011**, *82*, 899–904.
52. De oliveira, T.H.; Ginsberg, M.R.; Cooper, S.; Nowacki, A.; Rezai, A.; Deogaonkar, M.; Machado, A.G. Long-term effects of deep brain stimulation for essential tremor with subjective and objective quantification via mailed-in questionnaires. *Stereotact Funct. Neurosurg.* **2012**, *90*, 394–400.
53. Favilla, C.G.; Ullman, D.; Wagle shukla, A.; Foote, K.D.; Jacobson, C.E.; Okun, M.S. Worsening essential tremor following deep brain stimulation: Disease progression versus tolerance. *Brain* **2012**, *135*, 1455–1462.
54. Nazzaro, J.M.; Pahwa, R.; Lyons, K.E. Long-term benefits in quality of life after unilateral thalamic deep brain stimulation for essential tremor. *J. Neurosurg.* **2012**, *117*, 156–161.
55. Chang, W.S.; Chung, J.C.; Kim, J.P.; Chang, J.W. Simultaneous thalamic and posterior subthalamic electrode insertion with single deep brain stimulation electrode for essential tremor. *Neuromodulation* **2013**, *16*, 236–243.
56. Shih, L.C.; Lafaver, K.; Lim, C.; Papavassiliou, E.; Tarsy, D. Loss of benefit in VIM thalamic deep brain stimulation (DBS) for essential tremor (ET): How prevalent is it? *Parkinsonism Relat. Disord.* **2013**, *19*, 676–679.
57. Baizabal-Carvallo, J.F.; Kagnoff, M.N.; Jimenez-shahed, J.; Fekete, R.; Jankovic, J. The safety and efficacy of thalamic deep brain stimulation in essential tremor: 10 years and beyond. *J. Neurol. Neurosurg Psychiatry* **2014**, *85*, 567–572.
58. Cagnan, H.; Brittain, J.S.; Little, S.; Foltynie, T.; Limousin, P.; Zrinzo, L.; Hariz, M.; Joint, C.; Fitzgerald, J.; Green, A.; et al. Phase dependent modulation of tremor amplitude in essential tremor through thalamic stimulation. *Brain* **2013**, *136*, 3062–3075.
59. Pedrosa, D.J.; Reck, C.; Florin, E.; Pauls, M.; Maarouf, M.; Wojtecki, L.; Dafsari, S.H.; Sturm, V.; Schnitzler, A.; Fink, G.R.; et al. Essential tremor and tremor in Parkinson’s disease are associated with distinct 'tremor clusters' in the ventral thalamus. *Exp. Neurol.* **2012**, *237*, 435–443.
60. Ramirez-Zamora, A.; Boggs, H.; Pilitsis, J.G. Reduction in DBS frequency improves balance difficulties after thalamic DBS for essential tremor. *J. Neurol. Sci.* **2016**, *367*, 122–127.
61. Børretzen, M.N.; Bjerknes, S.; Sæhle, T.; Skjelland, M.; Skogseid, I.M.; Toft, M.; Dietrichs, E. Long-term follow-up of thalamic deep brain stimulation for essential tremor—patient satisfaction and mortality. *BMC Neurol.* **2014**, *14*, 120.
62. Coenen, V.A.; Allert, N.; Paus, S.; Kronenbürger, M.; Urbach, H.; Mädler, B. Modulation of the cerebello-thalamo-cortical network in thalamic deep brain stimulation for tremor: A diffusion tensor imaging study. *Neurosurgery* **2014**, *75*, 657–669.
63. Ehlen, F.; Schoenecker, T.; Kühn, A.A.; Klostermann, F. Differential effects of deep brain stimulation on verbal fluency. *Brain Lang.* **2014**, *134*, 23–33.

64. Groppa, S.; Herzog, J.; Falk, D.; Riedel, C.; Deuschl, G.; Volkmann, J. Physiological and anatomical decomposition of subthalamic neurostimulation effects in essential tremor. *Brain* **2014**, *137*, 109–121.
65. Mücke, D.; Becker, J.; Barbe, M.T.; Meister, I.; Liebhart, L.; Roettger, T.B.; Dembek, T.; Timmermann, L.; Grice, M. The effect of deep brain stimulation on the speech motor system. *J. Speech Lang. Hear. Res.* **2014**, *57*, 1206–1218.
66. Pedrosa, D.J.; Auth, M.; Pauls, K.A.; Runge, M.; Maarouf, M.; Fink, G.R.; Timmermann, L. Verbal fluency in essential tremor patients: The effects of deep brain stimulation. *Brain Stimul.* **2014**, *7*, 359–364.
67. Huss, D.S.; Dallapiazza, R.F.; Shah, B.B.; Harrison, M.B.; Diamond, J.; Elias, W.J. Functional assessment and quality of life in essential tremor with bilateral or unilateral DBS and focused ultrasound thalamotomy. *Mov. Disord.* **2015**, *30*, 1937–1943.
68. Becker, J.; Barbe, M.T.; Hartinger, M.; Dembek, T.A.; Pochmann, J.; Wirths, J.; Allert, N.; The effect of uni- and bilateral thalamic deep brain stimulation on speech in patients with essential tremor: Acoustics and intelligibility. *Neuromodulation* **2017**, *20*, 223–232.
69. Ehlen, F.; Vonberg, I.; Kühn, A.A.; Klostermann, F. Effects of thalamic deep brain stimulation on spontaneous language production. *Neuropsychologia* **2016**, *89*, 74–82.
70. Fytagoridis, A.; Åström, M.; Samuelsson, J.; Blomstedt, P. Deep brain stimulation of the caudal zona incerta: Tremor control in relation to the location of stimulation fields. *Stereotact Funct. Neurosurg.* **2016**, *94*, 363–370.
71. Gibson, W.S.; Jo, H.J.; Testini, P.; Cho, S.; Felmlee, J.P.; Welker, K.M.; Klassen, B.T.; Min, H.K.; Lee, K.H. Functional correlates of the therapeutic and adverse effects evoked by thalamic stimulation for essential tremor. *Brain* **2016**, *139*, 2198–2210.
72. Reich, M.M.; Brumberg, J.; Pozzi, N.G.; Marotta, G.; Roothans, J.; , Åström, M.; Musacchio, T.; Lopiano, L.; Lanotte, M.; Lehrke, R.; et al. Progressive gait ataxia following deep brain stimulation for essential tremor: Adverse effect or lack of efficacy? *Brain* **2016**, *139*, 2948–2956.
73. Allert, N.; Barbe, M.T.; Timmermann, L.; Coenen, V.A. Rapid battery depletion and loss of therapy due to a short circuit in bipolar DBS for essential tremor. *Acta Neurochir.* **2017**, *159*, 795–798.
74. Cagnan, H.; Pedrosa, D.; Little, S.; Pogosyan, A.; Cheeran, B.; Aziz, T.; Green, A.; Fitzgerald, J.; Foltynie, T.; Limousin, P.; et al.. Stimulating at the right time: Phase-specific deep brain stimulation. *Brain* **2017**, *140*, 132–145.
75. Chen, T.; Mirzadeh, Z.; Chapple, K.; Lambert, M.; Dhall, R.; Ponce, F.A. "Asleep" deep brain stimulation for essential tremor. *J. Neurosurg.* **2016**, *124*, 1842–1849.
76. Chockalingam, A.; Boggs, H.; Prusik, J.; Ramirez-Zamora, A.; Feustel, P.; Belasen, A.; Youn, Y.; Fama, C.; Haller, J.; Pilitsis, J. Evaluation of quantitative measurement techniques for head tremor with thalamic deep brain stimulation. *Neuromodulation* **2017**, *20*, 464–470.
77. Cury, R.G.; Fraix, V.; Castrioto, A.; Pérez-Fernández, M.A.; Krack, P.; Chabardes, S.; Seigneuret, E.; Alho, E.J.L.; Benabid, A.L.; Moro, E. Thalamic deep brain stimulation for tremor in Parkinson disease, essential tremor, and dystonia. *Neurology* **2017**, *89*, 1416–1423.
78. Ehlen, F.; Vonberg, I.; Tiedt, H.O.; Horn, A.; Fromm, O.; Kühn, A.A.; Klostermann, F. Thalamic deep brain stimulation decelerates automatic lexical activation. *Brain Cogn.* **2017**, *111*, 34–43.
79. Fiechter, M.; Nowacki, A.; Oertel, M.F.; Fichtner, J.; Debove, I.; Lachenmayer, M.L.; Wiest, R.; Bassetti, C.L.; Raabe, A.; Kaelin-Lang, A.; et al. Deep brain stimulation for tremor: Is there a common structure? *Stereotact Funct. Neurosurg.* **2017**, *95*, 243–250.
80. Rezaei Haddad, A.; Samuel, M.; Hulse, N.; Lin, H.Y.; Ashkan, K. Long-term efficacy of constant current deep brain stimulation in essential tremor. *Neuromodulation* **2017**, *20*, 437–443.
81. Holslag, J.A.H.; Neef, N.; Beudel, M.; ; Drost, G.; Oterdoom, D.L.M.; Kremer, N.I.; van Laar, T.; van Dijk, J.M.C. Deep brain stimulation for essential tremor: A comparison of targets. *World Neurosurg.* **2018**, *110*, e580–e584.
82. King, N.K.K.; Krishna, V.; Sammartino, F.; Bari, A.; Reddy, G.D.; Hodaie, M.; Kalia, S.K.; Fasano, A.; Munhoz, R.P.; Lozano, A.M.; et al. Anatomic targeting of the optimal location for thalamic deep brain stimulation in patients with essential tremor. *World Neurosurg.* **2017**, *107*, 168–174.
83. Wharen, R.E.; Okun, M.S.; Guthrie, B.L.; Uitti, R.J.; Larson, P.; Foote, K.; Walker, H.; Marshall, F.J.; Schwalb, J.; Ford, B.; et al. Thalamic DBS with a constant-current device in essential tremor: A controlled clinical trial. *Parkinsonism Relat. Disord.* **2017**, *40*, 18–26.

84. Akram, H.; Dayal, V.; Mahlknecht, P.; Georgiev, D.; Hyam, J.; Foltynie, T.; Limousin, P.; de Vita, E.; Jahanshahi, M.; Ashburner, J.; et al. Connectivity derived thalamic segmentation in deep brain stimulation for tremor. *Neuroimage Clin.* **2018**, *18*, 130–142.
85. De jesus, S.; Almeida, L.; Shahgholi, L.; Martinez-Ramirez, D.; Roper, J.; Hass, C.J.; Akbar, U.; Shukla, A.W.; Raike, R.S.; Okun, M.S. Square biphasic pulse deep brain stimulation for essential tremor: The BiP tremor study. *Parkinsonism Relat. Disord.* **2018**, *46*, 41–46.
86. Mücke, D.; Hermes, A.; Roettger, T.B.; Becker, J.; Niemann, H.; Dembek, T.A.; Timmermann, L.; Visser-Vandewalle, V.; Fink, G.R.; Grice, M.; et al. The effects of Thalamic Deep Brain Stimulation on speech dynamics in patients with Essential Tremor: An articulographic study. *PLoS ONE* **2018**, *13*, e0191359.

© 2020 by the authors. Submitted for possible open access publication under the terms and conditions of the Creative Commons Attribution (CC BY) license (http://creativecommons.org/licenses/by/4.0/).
